# Pharmacokinetics, pharmacodynamics, and safety of verinurad with and without allopurinol in healthy Asian, Chinese, and non‐Asian participants

**DOI:** 10.1002/prp2.929

**Published:** 2022-05-20

**Authors:** Susanne Johansson, David Han, Thomas Hunt, Karin Björck, Delia Florica, Michael Gillen, Jesse Hall, Fredrik Erlandsson

**Affiliations:** ^1^ Clinical Pharmacology & Quantitative Pharmacology Clinical Pharmacology & Safety Sciences AstraZeneca BioPharmaceuticals Research and Development Gothenburg Mölndal Sweden; ^2^ Parexel Early Phase Clinical Unit Los Angeles Glendale California USA; ^3^ PPD Development Austin Texas USA; ^4^ Biometrics CVRM AstraZeneca BioPharmaceuticals Research and Development Gothenburg Mölndal Sweden; ^5^ Patient Safety AstraZeneca BioPharmaceuticals Research and Development Gothenburg Sweden; ^6^ Formerly of AstraZeneca BioPharmaceuticals Research and Development Gaithersburg Maryland USA; ^7^ Formerly of Ardea Biosciences, Inc San Diego California USA; ^8^ CVRM Late Clinical AstraZeneca BioPharmaceuticals Research and Development Gothenburg Mölndal Sweden

**Keywords:** allopurinol, pharmacokinetics, Phase 1, safety, verinurad

## Abstract

Verinurad is a selective inhibitor of uric acid transporter 1 (URAT1). Here, we assessed the safety, pharmacokinetics, and pharmacodynamics of verinurad + allopurinol and verinurad monotherapy in healthy participants. Studies 1 (NCT03836599) and 2 (NCT02608710) were randomized Phase 1 studies. In Study 1, 12 healthy Asian participants received 24 mg verinurad + 300 mg allopurinol or placebo, and 9 healthy Chinese participants received 12 mg verinurad + 300 mg allopurinol. In Study 2, 24 healthy non‐Asian male participants received 12 mg verinurad. Safety analyses included assessment of adverse events (AEs). Pharmacokinetic parameters included maximum concentration (C_max_) and area under plasma concentration‐time curve (AUC) over 24 h (AUC_τ_). Pharmacodynamic parameters included percentage change from baseline (day –1) in serum uric acid (sUA) and urinary uric acid (uUA). There were no serious AEs or deaths in either study. In Study 1, steady‐state geometric mean (gCV%) C_max_ and AUC_τ_ values of verinurad after 7 days’ dosing were 73.6 (29.0) ng/mL and 478 (18.4) ng·h/mL, respectively, in healthy Asian participants, and 42.0 (40.1) ng/mL and 264 (36.1) ng·h/mL, respectively, in healthy Chinese participants; in Study 2, gCV% values were 36.3 (36.5) ng/mL and 271 (31.0) ng·h/mL, respectively. sUA decreased and uUA excretion increased compared with baseline following verinurad + allopurinol (Study 1) or verinurad (Study 2). When accounting for dose, the steady‐state pharmacokinetics of verinurad following multiple dosing were comparable between healthy Asian and Chinese participants and healthy non‐Asian participants. Verinurad treatments were well tolerated, including at higher verinurad exposures than previously evaluated after repeated dosing.

## Graphical abstract

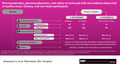

**Abbreviations**
AE: adverse event
ALT: alanine aminotransferase
AUC: area under plasma concentration‐time curve
AUC_τ_
: area under the curve over 24 hours
BMI: body mass index
CKD: chronic kidney disease
CL/F: apparent oral clearance
C_max_
: maximum concentration
ECG: electrocardiogram
FEUA: fractional excretion of uric acid
gCV%: geometric coefficient of variation
HFpEF: heart failure with preserved ejection fraction
NA: not applicable
QD: once daily
Rac: accumulation ratio
SAE: serious adverse event
SD: standard deviation
sUA: serum uric acid
t_½_λz: half‐life associated with terminal slop of a semi‐logarithmic concentration‐time curve
Tmax: time to reach Cmax
UA: uric acid
URAT1: UA transporter 1
uUA: urinary uric acid
XOI: xanthine oxidase inhibitor

## 1 INTRODUCTION

High levels of serum uric acid (sUA) are observed in chronic kidney disease (CKD) and heart failure with preserved ejection fraction (HFpEF) and may constitute either a contributing cause or risk factor for the diseases.^1^, ^2^, ^3^, ^4^ Consistent associations between sUA and CKD development and rapid progression^5^, ^6^, ^7^, ^8^ challenge the concept that increased sUA in patients with CKD should be viewed solely as a biomarker of decreased renal uric acid (UA) excretion. In HFpEF, an association between hyperuricemia and clinical outcomes, including all‐cause hospitalization, has also been reported.^1^, ^2^

Currently available therapies for the correction of sUA in hyperuricemia include xanthine oxidase inhibitors (XOI) such as allopurinol and febuxostat, which inhibit UA production, and UA transporter 1 (URAT1) inhibitors such as probenecid, benzbromarone, and lesinurad, which decrease sUA levels by inhibiting its reabsorption and increasing renal excretion of UA.^9^, ^10^, ^11^

Verinurad is a novel, selective, highly potent inhibitor of URAT1,^12^ which is in development for CKD and HFpEF. Verinurad shows dose‐proportional exposure (maximum observed plasma concentration [C_max_] and area under the curve [AUC]) up to 40 mg following a single dose^13^ and 15 mg following multiple once‐daily (QD) doses,^14^ and minimal accumulation (~1.2‐fold for C_max_ and 1.3‐fold for AUC) after QD doses.^14^ In a Phase 2a, randomized, double‐blind, placebo‐controlled trial (NCT03118739), the combination of verinurad + febuxostat lowered sUA and was well tolerated in patients with type 2 diabetes, albuminuria, and hyperuricemia.^15^ Based on the effects on albuminuria noted in Phase 2a, the combination of verinurad + allopurinol is being evaluated in 2 separate Phase 2b studies in patients with CKD (SAPPHIRE; NCT03990363) and HFpEF (AMETHYST; NCT04327024).

It is known that the pharmacokinetics of verinurad are dependent on renal function. In a single‐dose pharmacokinetics study in participants with varying degrees of renal impairment, verinurad C_max_ increased by 53%, 73%, and 128% and AUC increased by 24%, 148%, and 130% in participants with mild, moderate, and severe renal impairment, respectively, compared with normal renal function.^16^ Therefore, it is likely that patients with CKD will have verinurad exposure in the upper range or above what has previously been observed in healthy participants after repeat dosing. In addition, verinurad exposure (C_max_ and AUC) was comparable in healthy Japanese and non‐Asian participants when corrected for body weight.^14^ Whereas in 2 separate Phase 2 studies in patients with gout – 1 Japanese study and 1 Western study – verinurad exposure was >2‐fold higher than in Western patients.^17^ Therefore, we performed 2 Phase 1 studies which assessed the safety, pharmacokinetics, and pharmacodynamics of verinurad + allopurinol in healthy Asian and Chinese participants (Study 1; NCT03836599) and verinurad monotherapy in healthy non‐Asian participants (Study 2; NCT02608710). The specific aims of these studies in healthy participants were to: (1) assess high verinurad exposures with repeat dosing using the same type of formulation as is being used in the Phase 2b CKD and HFpEF studies, and (2) to provide additional data of treatment with verinurad in Asian participants (Study 1) compared with non‐Asian participants (Study 2).

## 2 MATERIALS AND METHODS

### 2.1 Study design

**FIGURE 1:**
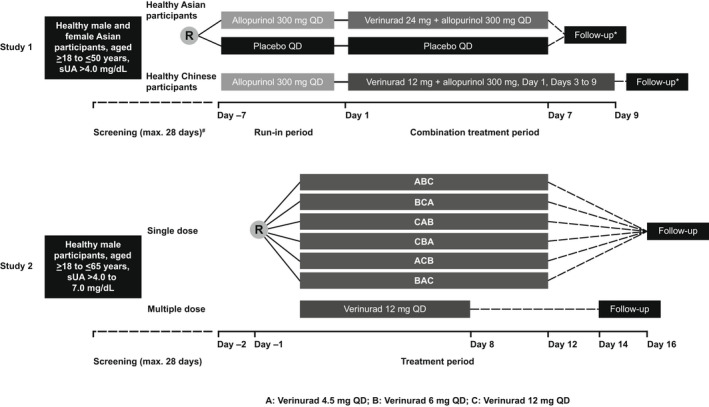
Design of Study 1 and Study 2. Abbreviations: QD, once daily; R, randomization; sUA, serum uric acid. *Participants returned for the follow‐up visit within 7–14 days of discharge from the clinical unit. ^#^The screening visit (Visit 1) was permitted to be conducted over 1 or more days during the Screening Period

Study 1 was a randomized, placebo‐controlled, Phase 1 study consisting of two cohorts. Cohort 1 was randomized, double‐blind, and placebo‐controlled and consisted of 12 healthy Asian participants, of whom <50% were Chinese. Cohort 2 was open‐label, consisting of 9 healthy Chinese participants. Each cohort underwent a screening period; a 7‐day run‐in period; a combination treatment period with verinurad + allopurinol for 7 days; and a follow‐up visit (Figure 1). The 7‐day run‐in period with 300 mg allopurinol was intended to decrease the risk of allopurinol‐induced toxicity; allopurinol administration was discontinued if skin rash or signs of allergic reaction appeared. Healthy Asian participants were randomized 3:1 on day –7 to 300 mg allopurinol or placebo QD. In the combination treatment period, participants receiving allopurinol during the run‐in period received 24 mg verinurad + 300 mg allopurinol QD during days 1 to 7, and participants receiving placebo continued on placebo. Healthy Chinese participants received 300 mg allopurinol QD during the run‐in period. In the combination treatment period, participants received 12 mg verinurad + 300 mg allopurinol QD on day 1, no dosing on day 2, and continued dosing on days 3 to 9.

Study 2 was a Phase 1, randomized, open‐label study comprising of three parts. Parts 1 and 2 assessed the pharmacokinetics and pharmacodynamics of single and multiple doses of verinurad, respectively (Figure 1). Part 3 assessed the effect of food on the pharmacokinetics and pharmacodynamics of verinurad and is not reported further herein. In the single‐dose assessment, 16 participants were randomized 1:1:1:1:1:1 into 1 of 6 treatment sequences with a single dose of 4.5, 6, or 12 mg verinurad, administered on days 1, 5, and 9 (Figure 1). In the multiple‐dose assessment, 8 participants received 12 mg verinurad QD on days 1 to 7.

In both studies, participants received verinurad capsules with an extended‐release profile. On the days of pharmacokinetic sampling, treatment was given in fasting state.

### 2.2 Participants

Eligible participants in Study 1 were healthy males and females who at screening were 18 to 50 years of age, had sUA level >4.0 mg/dL, body mass index (BMI) 18 to 30 kg/m^2^, and 50 to 100 kg bodyweight. A participant was considered Asian if the participant and both parents were part of the original peoples of the Far East, Southeast Asia, or the Indian subcontinent (including, for example, Cambodia, China, India, Japan, Korea, Malaysia, Pakistan, the Philippine Islands, Thailand, and Vietnam). A participant was considered Chinese if both parents and all grandparents were Chinese, and the participant was born in China and had not lived outside of China for more than 10 years.

For Study 2, eligible participants were healthy males who at screening were 18 to 65 years of age, had sUA level 4.0 to 7.0 mg/dL, BMI 18 to 40 kg/m^2^, and ≥50 kg bodyweight. There were no pre‐specified study inclusion criteria related to race or ethnicity; however, no Asian participants were enrolled in the study and, therefore, the cohort is referred to as the non‐Asian cohort. Key exclusion criteria are listed in the supplemental material.

### 2.3 Safety evaluation

Analysis of safety was based on adverse events (AEs), laboratory assessments (including hematology, clinical chemistry, urinalysis, and coagulation), vital signs (systolic and diastolic blood pressure, and pulse rate), electrocardiogram (ECG), and physical examination. AEs were coded using the Medical Dictionary for Regulatory Activities. In Study 1, a treatment‐emergent AE was defined as an AE with onset (start date/time) after the first dose of study drug in the run‐in period (day –7). In Study 2, AEs were recorded from the time the participant signed the informed consent through to the follow‐up visit. ECGs were obtained after the participant rested in the supine position. Digital ECG analysis in Study 1 was performed at the AstraZeneca ECG Center.

Safety was assessed in the safety analysis set of each study, comprising all participants who received ≥1 dose of study drug. Continuous variables are summarized using descriptive statistics, and categorical variables are summarized as frequency and proportion.

### 2.4 Pharmacokinetic and pharmacodynamic evaluation

#### 2.4.1 Sample collection and analytical methods

Plasma concentration measurements and serum and urine samples were obtained as described in Supplemental Table S1.

Plasma samples for pharmacokinetic analysis of verinurad, allopurinol, and oxypurinol in Study 1 were analyzed by Covance Bioanalytical Services, LLC (Indianapolis, IN, USA) and plasma samples for verinurad in Study 2 were analyzed by Ardea Biosciences, Inc. (San Diego, CA, USA), using methods validated according to the US Food and Drug Administration. Verinurad plasma samples were analyzed using similar methodologies as described by Shen et al.^13^ with lower and upper limits of quantification of 0.100 ng/mL and 40.0 ng/mL, respectively. The ability to dilute overrange samples was also confirmed. Intra‐ and inter‐run accuracy (% nominal) and precision (% CV) were in the ranges of 94.6–111% and ≤12.5%, respectively. The average mean recovery of verinurad and its [D_6_] stable labelled internal standard was 110% and 105%, respectively. Allopurinol and oxypurinol plasma samples were analyzed using similar methodologies as described by Kankam et al.^18^ with lower and upper limits of quantification of 25.0 ng/mL to 2000 ng/mL for allopurinol and 100 to 25 000 ng/mL for oxypurinol, respectively. The ability to dilute overrange samples was also confirmed. Intra‐ and inter‐run accuracy (% nominal) and precision (% CV) were in the ranges of 91.7–106% and ≤10.3%, respectively. The overall mean recovery of allopurinol and oxypurinol ranged from 88.5–91.8%, and the mean recovery of their stable labelled [^13^C_3_^15^N_3_] and [^13^C_2_^15^N] internal standards were 87.8% and 85.6%, respectively.

#### 2.4.2 Pharmacokinetic and pharmacodynamic analysis

Key pharmacokinetic parameters across both studies included AUC and AUC over 24 h (AUC_τ_), apparent oral clearance (CL/F), C_max_ and time to reach C_max_ (t_max_), half‐life (t_½_λz), and accumulation ratio (R_ac_) for C_max_ and AUC_τ_. t_½_λz was calculated as (ln2)/λz, where λz was estimated by log‐linear least squares regression of the terminal part of the concentration–time curve.

Key pharmacodynamic parameters included time‐matched percentage change from baseline (day –1) at each timepoint in sUA concentration, maximum observed percentage change from baseline (time‐matched, day –1) in sUA concentration (E_max,CB_) and time to E_max,CB_, amount of UA recovered in urine (Ae_ur_), and fractional excretion of UA (FEUA). Time‐matched changes were analyzed due to the diurnal variation in sUA frequently seen in participants treated with URAT1 inhibitors.^14^

Pharmacokinetics and pharmacodynamics were assessed in all participants who received ≥1 dose of study drug and had ≥1 corresponding assessment. Pharmacokinetic parameters were derived using noncompartmental methods with Phoenix^®^ WinNonlin^®^ version 6.3 or higher (Pharsight Corporation, Mountain View, CA, USA) and/or SAS^®^ version 9.2 (SAS Institute, Inc., Cary, NC, USA). Statistics for pharmacodynamics were calculated using SAS^®^ version 9.2 or higher. Data are summarized using number and percentage of participants, median and range, geometric mean with geometric coefficient of variation (gCV%), or arithmetic mean and standard deviation (SD). In Study 1, analyses of pharmacodynamic variables were performed on log‐transformed values.

## 3 RESULTS

### 3.1 Participants

In Study 1, 22 participants were randomized. Nine healthy Asian participants received allopurinol and 4 received placebo during the run‐in period; all but 1 participant completed the run‐in period and were randomized to 24 mg verinurad + 300 mg allopurinol (n = 9) or matching placebo (n = 3). Nine healthy Chinese participants received allopurinol during the run‐in period and 12 mg verinurad + 300 mg allopurinol in the combination treatment period.

In Study 2, 24 healthy non‐Asian participants were randomized. In the single‐dose assessment, 16 participants were randomized to 1 of 6 verinurad treatment sequences (Figure 1): ABC n = 2, BCA n = 3, CAB n = 3, CBA n = 3, ACB n = 3, BAC n = 2. In the multiple‐dose assessment, 8 participants received 12 mg verinurad QD. One participant from the multiple‐dose assessment withdrew from the study, after receiving 12 mg verinurad QD on days 1 to 7 as planned.

Baseline demographics and characteristics of participants in both studies are summarized in Table 1.

**TABLE 1:** Baseline demographic characteristics of participants in Study 1 and Study 2

	Study 1			Study 2	
	Healthy Asian participants		Healthy Chinese participants	Single‐dose assessment	Multiple‐dose assessment
	24 mg verinurad + 300 mg allopurinol (n = 9)	Placebo (n = 4)	12 mg verinurad + 300 mg allopurinol (n = 9)	Verinurad (n = 16)	12 mg verinurad (n = 8)
Sex, n (%)					
Male	8 (88.9)	3 (75.0)	9 (100.0)	16 (100.0)	8 (100.0)
Age, years					
Mean (SD)	39.8 (6.7)	33.8 (5.0)	38.2 (6.9)	38 (9.4)	33 (7.1)
Race, n (%)					
Asian	9 (100.0)	4 (100.0)	9 (100.0)	0	0
White	0	0	0	10 (62.5)	4 (50.0)
Black or African American	0	0	0	6 (37.5)	4 (50.0)
Ethnicity, n (%)					
Chinese	1 (11.1)	2 (50.0)	9 (100.0)	0	0
Chinese and Filipino	1 (11.1)	0	0	0	0
Chinese, Taiwanese	1 (11.1)	0	0	0	0
Filipino	0	1 (25.0)	0	0	0
Japanese	2 (22.2)	0	0	0	0
Korean	1 (11.1)	1 (25.0)	0	0	0
Korean, Japanese	1 (11.1)	0	0	0	0
Singapore	1 (11.1)	0	0	0	0
Thai	1 (11.1)	0	0	0	0
Hispanic or Latino	0	0	0	6 (37.5)	3 (37.5)
Not Hispanic or Latino	–	–	–	10 (62.5)	5 (62.5)
Height, cm					
Mean (SD)	174.1 (10.0)	169.8 (10.7)	172.6 (7.9)	173.6 (6.3)	170.4 (4.4)
Weight, kg					
Mean (SD)	75.6 (8.3)	74.5 (12.9)	73.1 (12.5)	84.3 (13.2)	81.6 (7.1)
BMI, kg/m^2^					
Mean (SD)	25.0 (2.8)	25.8 (3.5)	24.4 (2.5)	28.0 (4.1)	28.1 (1.8)
Serum uric acid, mg/dL					
Mean (SD)^a^	6.31 (1.2)	7.40 (3.2)	5.64 (0.8)	5.6 (0.8)	6.0 (0.8)

Note

Abbreviations: BMI, body mass index; SD, standard deviation.

^a^ Serum uric acid values are from screening, i.e., day –8 in Study 1 (before run‐in with allopurinol) and day –1 (–24 h) in Study 2.

### 3.2 Safety and tolerability

#### 3.2.1 Study 1

In healthy Asian participants, 2 (22.2%) participants receiving 24 mg verinurad + 300 mg allopurinol reported 4 AEs and 1 (33.3%) participant receiving placebo reported 1 AE in the combination treatment period (Table 2, Supplemental Table S2). In healthy Chinese participants, 5 (55.6%) participants receiving 12 mg verinurad + 300 mg allopurinol reported 11 AEs in the combination treatment period (Table 2, Supplemental Table S2). All AEs during the combination treatment period were mild in severity, and there were no serious AEs, deaths, or AEs leading to discontinuation of study drug (Table 2).

**TABLE 2:** Study 1: Summary of AEs with the combination of verinurad + allopurinol

	Healthy Asian participants				Healthy Chinese participants	
	24 mg verinurad + 300 mg allopurinol (n = 9)		Placebo (n = 3)		12 mg verinurad + 300 mg allopurinol (n = 9)	
	n (%)	Events	n (%)	Events	n (%)	Events
All AEs	2 (22.2)	4	1 (33.3)	1	5 (55.6)	11
AE severity						
Mild	2 (22.2)	4	1 (33.3)	1	5 (55.6)	11
Moderate	0	0	0	0	0	0
Severe	0	0	0	0	0	0
AEs related to study drug	2 (22.2)	3	1 (33.3)	1	4 (44.4)	7
Serious AEs	0	0	0	0	0	0
Death	0	0	0	0	0	0
AEs leading to study drug discontinuation	0	0	0	0	0	0

Note

Events that emerged during the combination treatment period. AE severity was assessed using the following scale: mild: awareness of sign or symptom, but easily tolerated; moderate: discomfort sufficient to cause interference with normal activities; severe: incapacitating, with inability to perform normal activities.

Abbreviation: AE, adverse event.

AEs reported during the run‐in period are described in the supplemental material. One healthy Asian participant receiving placebo prematurely withdrew from the study due to an AE of clinically significant increases of transaminases (alanine aminotransferase [ALT] and aspartate aminotransferase), considered by the investigator to be of moderate intensity and related to placebo. The participant's ALT peaked at 311 IU/L; abnormal liver function tests were monitored over 1 month until near normalization. There were no other clinically significant findings in laboratory assessments.

No clinically significant findings were noted in vital signs or digital ECG variables (supplemental material), nor abnormal findings during physical examinations, in the active treatment groups.

#### 3.2.2 Study 2

Two healthy non‐Asian participants reported 2 AEs in the single‐dose assessment and 2 participants reported 3 AEs in the multiple‐dose assessment (Table 3). No AEs occurred in more than 1 participant, with the exception of diarrhea (2 participants; Supplemental Table S3). All AEs were mild in severity, with the exception of 2 moderate AEs (gastrointestinal motility disorder with 6 mg verinurad; rash pustular with 12 mg verinurad). There were no serious AEs, deaths, or AEs leading to withdrawal (Table 3).

**TABLE 3:** Study 2: Summary of AEs

	Single‐dose assessment						Multiple‐dose assessment	
	4.5 mg verinurad (n = 16)		6 mg verinurad (n = 16)		12 mg verinurad (n = 16)		12 mg verinurad QD (n = 8)	
	n (%)	Events	n (%)	Events	n (%)	Events	n (%)	Events
All AEs	0	0	1 (6.3)	1	1 (6.3)	1	2 (25.0)	3
AE severity								
Mild	0	0	0	0	1 (6.3)	1	1 (12.5)	2
Moderate	0	0	1 (6.3)	1	0	0	1 (12.5)	1
Severe	0	0	0	0	0	0	0	0
AEs possibly related to study drug	0	0	0	0	1 (6.3)	1	1 (12.5)	1
Serious AEs	0	0	0	0	0	0	0	0
Death	0	0	0	0	0	0	0	0
AEs leading to withdrawal	0	0	0	0	0	0	0	0

Note

AE severity was assessed using the Rheumatology Common Toxicity Criteria.

Abbreviations: AE, adverse event; QD, once daily.

No clinically significant findings were noted in laboratory parameters, vital signs, or 12‐lead ECG variables. Except for a resolving contusion reported at the discharge and follow‐up visit in 1 participant, there were no clinically significant findings in physical examinations.

### 3.3 Pharmacokinetics

#### 3.3.1 Study 1

**FIGURE 2:**
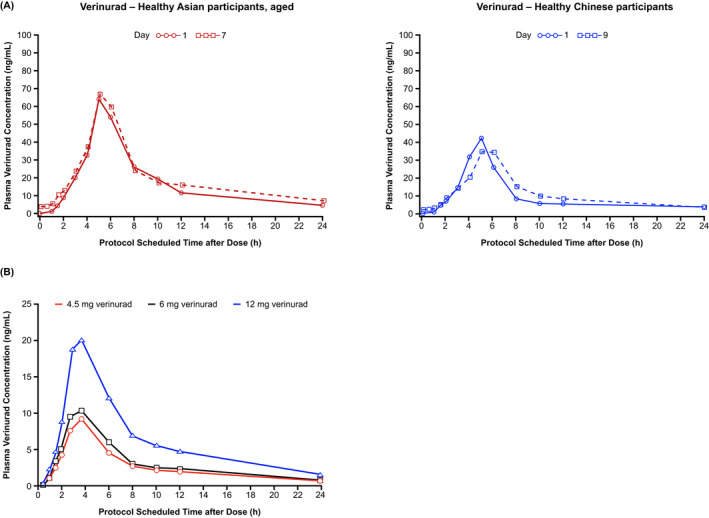
Geometric mean verinurad plasma concentration‐time profiles (A) following single and multiple dosing in healthy Asian and Chinese participants in Study 1 and (B) following single doses in healthy non‐Asian participants in Study 2. (A) Healthy Asian participants: 24 mg verinurad + 300 mg allopurinol once daily for 7 days. Healthy Chinese participants: 12 mg verinurad + 300 mg allopurinol on day 1 and then once daily on days 3–9. (B) Single‐dose assessment: participants were randomized in a 1:1:1:1:1:1 ratio into 1 of 6 treatment sequences with a single dose of 4.5, 6, or 12 mg verinurad, administered on days 1, 5, and 9

At steady state after 7 days’ QD dosing, the geometric means (gCV%) for C_max_ and AUC_τ_ of verinurad were 73.6 (29.0) ng/mL and 478 (18.4) ng·h/mL, respectively, in healthy Asian participants (24 mg verinurad), and 42.0 (40.1) ng/mL and 264 (36.1) ng·h/mL, respectively, in healthy Chinese participants (12 mg verinurad) (Table 4). Plasma concentrations of verinurad showed biphasic decline following C_max_ (Figure 2). Minimal accumulation of verinurad was seen after QD dosing based on R_ac_ C_max_ and R_ac_ AUC_τ_ in healthy Asian (1.03 and 1.15, respectively) and Chinese (0.959 and 1.23, respectively) participants (Table 4).

**TABLE 4:** Study 1: Summary of pharmacokinetic parameters for verinurad in healthy Asian and Chinese participants

Study Day	AUC (ng·h/mL)	AUC_τ_ (ng·h/mL)	CL/F (L/h)	C_max_ (ng/mL)	t_max_ (h)	t_½_λz (h)	R_ac_ AUC_τ_	R_ac_ C_max_
Verinurad: Healthy Asian participants								
Day 1	506 (24.8)	415 (25.7)	48.6 (11.3)	71.2 (34.9)	5.00 (4.98–6.02)	5.98 (1.33)	N/A	N/A
Day 7	N/A	478 (18.4)	50.9 (9.01)	73.6 (29.0)	5.00 (4.00–6.00)	N/A	1.15 (0.934–1.60)	1.03 (0.779–1.43)
Verinurad: Healthy Chinese participants								
Day 1	295 (39.9)	226 (42.7)	43.4 (16.2)	47.6 (50.2)	5.00 (4.00–6.00)	12.9 (3.08)	N/A	N/A
Day 9	N/A	264 (36.1)	47.9 (17.2)	42.0 (40.1)	5.50 (4.00–6.08)	6.27 (0.647)	1.23 (0.974–1.90)	0.959 (0.691–2.26)

Note

AUC, AUC_τ_, and C_max_, are geometric mean (gCV%); CL/F and t_½_λz are arithmetic mean (SD); t_max_ is median (range); and R_ac_ AUC_τ_ and R_ac_ C_max_ are geometric mean (range).

Abbreviations: AUC, area under plasma concentration‐time curve; AUC_τ_, AUC over a dosing interval (24 h); CL/F, apparent oral clearance; C_max_, maximum observed plasma concentration; gCV%, geometric coefficient of variation; R_ac_, accumulation ratio; SD, standard deviation; t_½_λz, half‐life associated with terminal slope of a semi‐logarithmic concentration‐time curve; t_max_, time to reach C_max_.

In both healthy Asian and Chinese participants, plasma concentration–time profiles of allopurinol and oxypurinol on the last day of 300 mg allopurinol QD dosing showed monophasic decline following C_max_ (Supplemental Figure S1). Minimal accumulation of allopurinol was seen after QD dosing based on R_ac_ C_max_ and R_ac_ AUC_τ_ in healthy Asian (1.00 and 0.967, respectively) and Chinese (0.787 and 0.932, respectively) participants (Supplemental Table S4). In both cohorts, the AUC_τ_ and C_max_ of oxypurinol were reduced on the last day of 300 mg allopurinol QD compared with day 1 (Supplemental Table S4).

#### 3.3.2 Study 2

Following a single dose of verinurad 4.5, 6, or 12 mg, geometric mean C_max_ ranged from 11.8 to 28.6 ng/mL and geometric mean AUC_τ_ ranged from 68.1 to 168 ng·h/mL (Table 5) and appeared to increase dose‐proportionally in healthy non‐Asian participants. Plasma concentrations of verinurad showed biphasic decline following C_max_ (Figure 2). In the multiple‐dose assessment, geometric means (gCV%) for C_max_ and AUC_τ_ of verinurad were 36.3 (36.5) ng/mL and 271 (31.0) ng∙hr/mL, respectively, following 12 mg verinurad QD on day 7 (Table 5). The mean R_ac_ C_max_ and R_ac_ AUC_τ_ values for verinurad on day 7 were 1.21 and 1.30, respectively (Table 5).

**TABLE 5:** Study 2: Summary of pharmacokinetic parameters for verinurad in healthy non‐Asian participants

	AUC (ng·h/mL)	AUC_τ_ (ng·h/mL)	CL/F (L/h)	C_max_ (ng/mL)	t_max_ (h)	t_½_λz (h)	R_ac_ AUC_τ_	R_ac_ C_max_
Single‐dose assessment								
4.5 mg	86.8 (31.3)	68.1 (33.6)	51.8 (27.9)	11.8 (64.0)	4.00 (2.00–6.00)	12.7 (76.5)	N/A	N/A
6 mg	102 (47.4)	80.8 (45.7)	58.8 (40.5)	13.4 (67.6)	4.00 (2.00–6.05)	13.6 (58.3)	N/A	N/A
12 mg	206 (41.1)	168 (39.1)	58.3 (44.4)	28.6 (76.5)	4.00 (2.00–6.00)	12.2 (67.7)	N/A	N/A
Multiple‐dose assessment								
Day 1	230 (18.6)	209 (18.6)	52.2 (20.2)	29.9 (32.2)	5.00 (2.00–6.00)	7.15 (25.8)	N/A	N/A
Day 7	N/A	271 (31.0)	44.2 (27.3)	36.3 (36.5)	6.00 (3.00–8.00)	10.9 (19.1)	1.30 (26.3)	1.21 (41.7)

Note

Abbreviations: AUC, area under plasma concentration‐time curve; AUC_τ_, AUC over a dosing interval (24 h); CL/F, apparent oral clearance; C_max_, maximum observed plasma concentration; gCV%, geometric coefficient of variation; R_ac_, accumulation ratio; t_½_λz, half‐life associated with terminal slope of a semi‐logarithmic concentration‐time curve; t_max_, time to reach C_max_.

Data shown are geometric mean (gCV%), except for t_max_ which is median (range).

### 3.4 Pharmacodynamics

#### 3.4.1 Study 1

**FIGURE 3:**
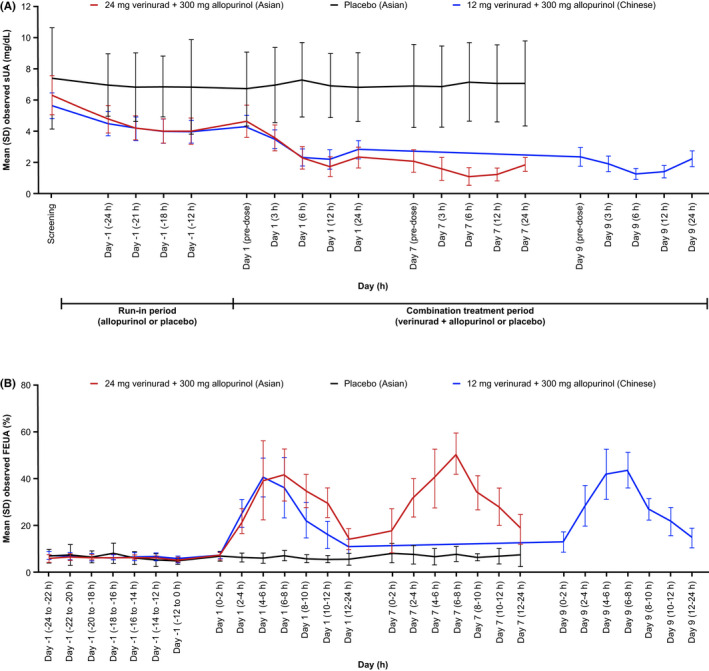
Study 1: Mean (SD) observed (A) sUA and (B) FEUA in healthy Asian and Chinese participants. Healthy Asian participants: 24 mg verinurad + 300 mg allopurinol or matching placebo once daily for 7 days. Healthy Chinese participants: 12 mg verinurad + 300 mg allopurinol on day 1 and then once daily on days 3–9. Baseline (day –1) represents sUA and FEUA following 7 days of 300 mg allopurinol during the run‐in period. In healthy Asian participants, sUA levels at screening (day –8) and baseline (day –1), respectively, were 6.31 mg/dL and 4.77 mg/dL with 24 mg verinurad + 300 mg allopurinol and 7.40 mg/dL and 6.97 mg/dL with matching placebo. In healthy Chinese participants, sUA levels at screening (day –8) and baseline (day –1), respectively, were 5.64 mg/dL and 4.49 mg/dL with 12 mg verinurad + 300 mg allopurinol. Abbreviations: FEUA, fractional excretion of uric acid; SD, standard deviation; sUA, serum uric acid

Levels of sUA at screening for the 2 cohorts are shown in Table 1. sUA was reduced from screening after 300 mg allopurinol QD for 7 days, with a mean percentage change on day 1 (pre‐dose) of −26.1% in healthy Asian participants and −23.5% in healthy Chinese participants. The combination of 12/24 mg verinurad + 300 mg allopurinol for 7 days further reduced sUA levels compared with day –1 (Figure 3A). In healthy Asian participants, mean E_max,CB_ of sUA with 24 mg verinurad + 300 mg allopurinol on day 1 was –58.3% and on day 7 was −73.9%, compared with day –1 (Supplemental Table S5). No clinically meaningful changes in sUA were seen in healthy Asian participants receiving placebo. In healthy Chinese participants, mean E_max,CB_ of sUA with 12 mg verinurad + 300 mg allopurinol on day 1 was −44.9% and on day 9 was −‍67.2%, compared with day –1 (Supplemental Table S5).

In healthy Asian participants, mean percentage change from baseline (day –1) of Ae_ur_ peaked at 4 to 6 h post‐dose on days 1 and 7 with 24 mg verinurad + 300 mg allopurinol; mean (range) percentage change was 375% (82–638%) on day 1 and 106% (−5 to 250%) on day 7 (Supplemental Figure S2). No clinically meaningful changes were seen in participants receiving placebo. In healthy Chinese participants, mean percentage change of Ae_ur_ peaked at 4 to 6 h post‐dose on day 1 and 2 to 4 h post‐dose on day 9 with 12 mg verinurad + 300 mg allopurinol; mean (range) percentage change of Ae_ur_ was 437% (223–1256%) on day 1 and 300% (−24 to 1531%) on day 9 (Supplemental Figure S2).

On the last day of dosing, FEUA in healthy Asian participants peaked at 6 to 8 h post‐dose with a mean FEUA of 50.7% with 24 mg verinurad + 300 mg allopurinol, and in healthy Chinese participants peaked at 6 to 8 h post‐dose with a mean FEUA of 43.7% with 12 mg verinurad + 300 mg allopurinol (Figure 3B).

#### 3.4.2 Study 2

**FIGURE 4:**
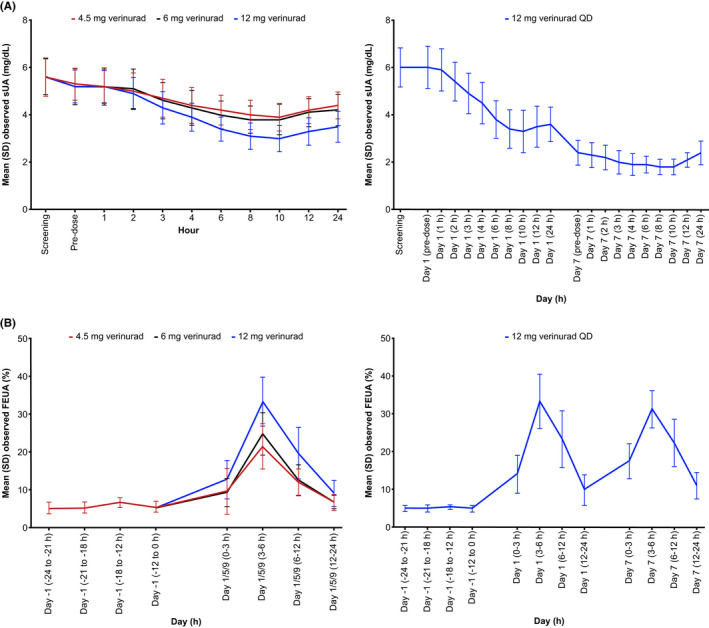
Study 2: Mean (SD) observed (A) sUA and (B) FEUA following single doses and multiple doses of verinurad in healthy non‐Asian participants. sUA levels at screening (day –1, –24 h) in the single‐dose and multiple‐dose assessments were 5.6 mg/dL and 6.0 mg/dL, respectively. Abbreviations: FEUA, fractional excretion of uric acid; QD, once daily; SD, standard deviation; sUA, serum uric acid

sUA decreased dose‐proportionally following 4.5, 6, and 12 mg verinurad in healthy non‐Asian participants (Figure 4A). Mean E_max,CB_ of sUA was −25.8%, −29.4%, and −42.2% with 4.5, 6, and 12 mg verinurad, respectively, compared with day –1 pre‐dose (Supplemental Table S5). In the multiple‐dose assessment, mean E_max,CB_ of sUA on day 1 was −46.5% and on day 7 was −70.9%, compared with day –1 pre‐dose (Supplemental Table S5).

In the single‐dose assessment, mean Ae_ur_ assessed over 24 h post‐dose increased dose‐proportionally. Mean Ae_ur_ peaked at 3 to 6 h post‐dose; mean Ae_ur_ following 4.5, 6, and 12 mg verinurad was 219%, 267%, and 338% higher, respectively, compared with day –1 (Supplemental Figure S3A). In the multiple‐dose assessment, mean Ae_ur_ peaked 3 to 6 h post‐dose following 12 mg verinurad QD on days 1 and 7; mean Ae_ur_ was 353% and 81.3% higher, respectively, compared with day –1 (Supplemental Figure S3B).

Following 4.5, 6, and 12 mg verinurad, FEUA peaked at 3 to 6 h post‐dose and increased dose‐proportionally; mean FEUA was 21.5%, 24.8%, and 33.3%, respectively (Figure 4B). In the multiple‐dose assessment, FEUA peaked at 3 to 6 h post‐dose on both days 1 and 7 following 12 mg verinurad QD; mean FEUA was 33.3% and 31.2%, respectively (Figure 4B).

## 4 DISCUSSION

In the present Phase 1 studies, we evaluated the safety, pharmacokinetics, and pharmacodynamics of verinurad + allopurinol in healthy Asian and Chinese participants, and verinurad alone in healthy non‐Asian participants.

The first aim of these studies was to assess high verinurad exposures with repeat dosing. In Study 1, higher verinurad exposures with repeat dosing than those assessed in earlier stage clinical studies of verinurad were achieved. Indeed, steady‐state C_max_ and AUC_τ_ values for verinurad of 73.6 ng/mL and 478 ng·h/mL, respectively, were achieved with 24 mg extended‐release verinurad + 300 mg allopurinol in healthy Asian participants. By comparison, among healthy Japanese male participants, C_max_ and AUC_τ_ values for verinurad of 38.2 ng/mL and 239 ng·h/mL, respectively, were observed on day 6 after QD dosing with 15 mg verinurad given as a modified‐release formulation,^14^ which has a lower bioavailability compared with the formulation used in Studies 1 and 2. To put this into perspective, increased verinurad exposures have been observed in participants with renal impairment receiving 15 mg verinurad, with an increased C_max_ of 73% and 128% and an increased AUC of 148% and 130% in participants with moderate and severe renal impairment, respectively, compared with normal renal function.^16^ Given the potential for higher verinurad exposure in the target patient population, i.e. those with reduced renal function,^16^ assessment of higher exposures of verinurad than previously assessed during repeated dosing was therefore warranted. We observed no safety or tolerability concerns with dosing of up to 24 mg verinurad + 300 mg allopurinol in healthy Asian participants. Overall, both studies suggest that the exposures of verinurad achieved in these 2 studies in healthy participants are tolerable.

The second aim of these studies was to provide additional data of treatment with verinurad in Asian participants. Previous verinurad studies among the Asian population have predominantly included Japanese participants.^14^, ^17^ Study 1 was performed to provide data for verinurad treatment in Asian participants regardless of ethnicity and assess a population consisting only of Chinese participants. When accounting for differences in dose, the steady‐state exposure of verinurad following QD dosing for 7 days was generally comparable between healthy Asian and Chinese participants (Study 1) and between healthy Asian and non‐Asian participants (Study 2). Accordingly, these findings suggest that Asian ethnicity does not impact verinurad pharmacokinetics.

Data from Study 2 (4.5–12 mg verinurad) and Study 1 (12 and 24 mg verinurad) support that verinurad exposure increases dose‐proportionally in the studied dose range, with minimal accumulation following multiple QD dosing, as seen previously with verinurad among healthy Japanese participants.^14^

In Study 1, comparison of day 1 and day 7/9 data suggests that verinurad co‐administration did not influence the exposure of allopurinol, which is consistent with the results from a drug–drug interaction study with verinurad and allopurinol among patients with gout;^18^ this study also showed that allopurinol does not influence the pharmacokinetics of verinurad.^18^ Oxypurinol exposure was lower on day 7/9 compared with day 1, which is to be expected following several days of co‐administration with verinurad.^18^

In both studies, healthy participants had baseline sUA levels near the upper limit of normal (i.e., ~6 mg/dL) at screening, and sUA decreased following treatment with verinurad monotherapy (Study 2) or verinurad + allopurinol (Study 1). In Study 2, sUA decreased dose‐proportionally between the studied doses of verinurad (4.5–12 mg), supporting dose‐dependent reductions in sUA observed previously.^14^, ^19^, ^20^ In Study 1, both studied verinurad doses (12 and 24 mg QD for 7 days) + 300 mg allopurinol had comparable mean E_max,CB_ of sUA, suggesting that the verinurad exposure achieved with 12 and 24 mg doses is within the upper part of the exposure–‍sUA relationship. Importantly, baseline in Study 1 was defined as the last day of treatment with allopurinol 300 mg for 7 days (day –1), and so E_max,CB_ values for sUA with verinurad + allopurinol represent changes following treatment with allopurinol 300 mg for 7 days. Therefore, changes in sUA after 7 days of verinurad + allopurinol were compared with screening values; these data suggest that 12 or 24 mg verinurad + 300 mg allopurinol will likely result in percent changes in sUA of approximately −80% in healthy participants. Overall, these data confirm our previous knowledge that high doses/exposure of verinurad in combination with an XOI are likely to normalize sUA levels among most patients with hyperuricemia.

Consistent with its mechanism of action as a selective reabsorption inhibitor of URAT1, FEUA increased following verinurad monotherapy (Study 2) or verinurad in combination with an XOI (Study 1). Increases in FEUA with verinurad among healthy non‐Asian participants in Study 2 were dose‐dependent, consistent with earlier findings using single and multiple doses of verinurad in healthy Japanese and non‐Asian participants.^14^

The Phase 1 studies presented here have several potential limitations. A direct within‐study comparison of race/ethnicity could not be performed as neither study included both healthy Asian and non‐Asian participants. However, a similar design and aim of the Phase 1 studies made a between‐study comparison possible. In Study 1, it is difficult to draw firm conclusions on the true effect of the pharmacodynamics of verinurad + allopurinol, as true baseline measurements, i.e., without either treatment, were not taken. Baseline was instead defined as the measurement after 7 days of 300 mg allopurinol QD. Although changes from screening (i.e., before allopurinol treatment) are interesting, the timing of the screening visit was not controlled using the same time‐matched criteria as for baseline. Finally, direct within‐study comparison of pharmacodynamics and safety could not be performed due to the impact of differences in treatment regimen (combination therapy of verinurad + allopurinol in Study 1 and verinurad monotherapy in Study 2) and differences in scales used to assess AE severity.

Phase 2 studies evaluating verinurad combined with 300 mg allopurinol are currently ongoing in patients with CKD and HFpEF. Studies 1 and 2 allowed the evaluation of verinurad at exposures expected to be seen in patients with impaired renal function after dosing of the highest dose currently being tested in Phase 2.

## 5 CONCLUSIONS

Verinurad treatments were well tolerated in both studies. The pharmacokinetics of verinurad after QD dosing were comparable in healthy Asian and Chinese participants and healthy non‐Asian participants. Further exploration of verinurad doses up to 24 mg in combination with an XOI in Phase 2 dose‐finding studies is underway.

## ETHICS STATEMENT

Both studies were performed in accordance with the ethical principles of the Declaration of Helsinki and the International Council for Harmonisation/Good Clinical Practice. The final study protocols, including the informed consent forms, as well as any amendments were approved by an independent Ethics Committee or Institutional Review Board (Study 1: Aspire IRB, Santee, CA, USA; Study 2: IntegReview IRB, Austin, TX, USA). The studies were conducted at Parexel Early Phase Clinical Unit Los Angeles, Glendale, CA, USA (Study 1) or PPD Phase 1 Clinic, Austin, TX, USA (Study 2).

## DISCLOSURE

S.J., K.B., D.F., and F.E. are employees of AstraZeneca. D.H. and T.H. declare no conflict of interests. M.G. is a former employee of and currently a consultant for AstraZeneca, and also owns stock in AstraZeneca. J.H. is a former employee of Ardea Biosciences, a subsidiary of AstraZeneca.

## AUTHOR CONTRIBUTIONS

S.J., F.E., and M.G. were involved in the study design and implementation. D.H. and T.H. were involved in the data collection. S.J., D.H., T.H., K.B., D.E., M.G., J.H. and F.E. were involved in the data analysis and interpretation. All authors critically reviewed the manuscript, approved the final version, and accept accountability for the overall work.

## STUDY PRINCIPAL INVESTIGATOR STATEMENT

The authors confirm that the PIs for this paper are Drs David Han and Thomas Hunt and that they had direct clinical responsibility for patients.

## PATIENT CONSENT STATEMENT

All participants provided written informed consent.

## Supporting information

Supplementary Material

## Data Availability

Data underlying the findings described in this manuscript may be obtained in accordance with AstraZeneca's data sharing policy described at https://astrazenecagroup‐dt.pharmacm.com/DT/Home.
