# Supplementary material for: Pharmacokinetics, pharmacodynamics, and safety of verinurad with and without allopurinol in healthy Asian, Chinese, and non‐Asian participants
Source: Pharmacol Res Perspect. 2022 May 20;10(3):e00929. doi: 10.1002/prp2.929 (PMC9121888; doi:10.1002/prp2.929)
Supplement: Supplementary file 1 — Supplementary Material [file PRP2-10-e00929-s001.docx]

# Supplemental Materials

## Inclusion Criteria and Exclusion Criteria

### Study 1

Additional inclusion criteria:

1. Females had a negative pregnancy test at the Screening Visit and day –7, were not lactating, and were:

- Of non-childbearing potential, confirmed at the Screening Visit by fulfilling one of the following criteria:
- Post-menopausal, defined as amenorrhea for at least 12 months or more following cessation of all exogenous hormonal treatments and follicle stimulating hormone levels in the post-menopausal range (>40 IU/mL).
- Documentation of irreversible surgical sterilization by hysterectomy, bilateral oophorectomy, or bilateral salpingectomy but not tubal ligation.
- OR if of childbearing potential were willing to use an acceptable method of contraception to avoid pregnancy for the entire study period.

Key exclusion criteria:

1. History or presence of gastrointestinal, hepatic or renal disease, or any other condition known to interfere with absorption, distribution, metabolism, or excretion of drugs.
2. Any clinically significant abnormalities in clinical chemistry, hematology, or urinalysis results, at the Screening Visit as judged by the Investigator.
3. Known carrier of the human leukocyte antigen B *58:01 allele.
4. Any clinically significant abnormal findings in vital signs at the Screening Visit, defined as any of the following:

- Systolic blood pressure (BP) <90 mmHg or >140 mmHg;
- Diastolic BP <50 mmHg or >90 mmHg;
- Heart rate <50 or >90 beats per minute.

1. Any clinically important abnormalities in rhythm, conduction, or morphology of the 12-lead resting electrocardiogram (ECG) and any clinically important abnormalities in the 12-lead ECG as considered by the Investigator that may interfere with the interpretation of QT interval corrected for heart rate using Fridericia’s formula (QTcF) interval changes, including abnormal ST-T wave morphology.
2. Any positive result on Screening for serum hepatitis B surface antigen, hepatitis C antibody (Ab), and human immunodeficiency virus (HIV) Ab.
3. History of severe allergy/hypersensitivity or ongoing clinically relevant important allergy/hypersensitivity, as judged by the Investigator, or history of hypersensitivity to drugs with a similar chemical structure or class as verinurad or allopurinol.

### Study 2

Key exclusion criteria:

1. Participant had a history or suspicion of kidney stones.
2. Participant had any gastrointestinal disorder that affected motility and/or absorption.
3. Participant had a history of malignancy with the exception of nonmelanoma skin cancer.
4. Participant had a clinically relevant intolerance or allergy to foods or drugs, or a known or suspected hypersensitivity to any ingredient in the investigational medicinal product (IMP).
5. Participant had clinically relevant abnormalities in BP, heart rate, or body temperature, per the Investigator’s judgement.
6. Participant had Screening clinical safety laboratory parameters (serum chemistry [other than serum creatinine and serum uric acid], hematology, coagulation, or urinalysis) that are outside the normal limits and are considered clinically significant by the Investigator.
7. Participant had a serum creatinine value above the upper limit of normal (ULN) at the Screening Visit.
8. Participant had clinically relevant abnormalities in 12-lead ECG, per the Investigator’s judgement.
9. Participant had a history of cardiac abnormalities as assessed during Screening, including abnormal and clinically relevant ECG changes.
10. Participant had positive serology to HIV-1 and HIV-2.
11. Participant had a positive test for active hepatitis B or hepatitis C infection.
12. Participant had received any strong or moderate enzyme-inducing drug or product within 2 months prior to day 1.

## Safety and Tolerability

### Study 1

During the run-in period, in healthy Asian participants, 1 participant in the verinurad + allopurinol group and 1 participant in the placebo group each reported 1 event (musculoskeletal pain and transaminases increased, respectively). In healthy Chinese participants, 2 participants in the verinurad + allopurinol group reported 4 events (diarrhea, lip blister, dry skin, pruritis). All but 1 of the adverse events were mild; there were no deaths or serious adverse events.

### Digital ECG Findings in Study 1

There were no clinically significant QTcF prolongations in the active treatment groups, ie, 24 mg verinurad + 300 mg allopurinol and 12 mg verinurad + 300 mg allopurinol. The categorical summary of QTcF values observed during the study did not show any participants with outlying QTcF values in the active treatment groups. There were no clinically significant changes from baseline in the other digitally analyzed ECG variables (pulse rate, onset of the QRS complex to the J point [QRS], QT, time between corresponding points on 2 consecutive R waves on ECG [RR], and heart rate) in the active treatment groups.

Supplemental Table S1 Sample collection for pharmacokinetic and pharmacodynamic assessments

|  | **Study 1** | | **Study 2** | |
| --- | --- | --- | --- | --- |
|  | **Healthy Asian participants** | **Healthy Chinese participants** | **Single-dose assessment** | **Multiple-dose assessment** |
| **Pharmacokinetics** | | | | |
| Plasma | Days 1 and 7: pre-dose, and 0.5, 1, 1.5, 2, 3, 4, 5, 6, 8, 10, 12, and 24 hours post-dose; Days 3 to 6: pre-dose | Days 1 and 9: pre dose, and 0.5, 1, 1.5, 2, 3, 4, 5, 6, 8, 10, 12, and 24 hours post-dose; Day 2: 36 hours post-first dose; Days 3 to 8: pre-dose | Days 1, 5, and 9: within 30 minutes pre-dose, and at 30 minutes and 1, 1.5, 2, 3, 4, 6, 8, 10, 12, 24, 36, 48, and 72 hours post-dose | Days 1 and 7: within 30 minutes pre-dose, and at 30 minutes and 1, 1.5, 2, 3, 4, 6, 8, 10, 12, and 24 hours post-dose |
| **Pharmacodynamics** | | | | |
| Serum for uric acid | Days 1 and 7: pre-dose, and 3, 6, 12, and 24 hours post-dose | Days 1 and 9: pre-dose, and 3, 6, 12, and 24 hours post-dose | Days 1, 5, and 9: within 30 minutes pre-dose, and 1, 2, 3, 4, 6, 8, 10, 12, and 24 hours post-dose | Days 1 and 7: within 30 minutes pre-dose, and 1, 2, 3, 4, 6, 8, 10, 12, and 24 hours post-dose |
| Urine collection for uric acid | Days 1 and 7: 0–2, 2–4, 4–6,  6–8, 8–10, 10–12, and  12–24 hours post-dose | Days 1 and 9: 0–2, 2–4, 4–6,  6–8, 8–10, 10–12, and  12–24 hours post-dose | Days 1, 5, and 9: 0–3, 3–6,  6–12, and 12–24 hours post-dose | Days 1 and 7: 0–3, 3–6, 6–12, and 12–24 hours post-dose |

Supplemental Table S2 Study 1: Summary of AEs with the combination of verinurad + allopurinol by system organ class and preferred term

| **System organ class, n (%) Preferred term** | **Healthy Asian participants** | | **Healthy Chinese participants** |
| --- | --- | --- | --- |
|  | **24 mg verinurad + 300 mg allopurinol (n = 9)** | **Placebo (n = 3)** | **12 mg verinurad +  300 mg allopurinol (n = 9)** |
| Gastrointestinal disorders | 2 (22.2) | 0 | 3 (33.3) |
| Diarrhea | 2 (22.2) | 0 | 0 |
| Abdominal pain | 0 | 0 | 1 (11.1) |
| Aphthous ulcer | 0 | 0 | 1 (11.1) |
| Constipation | 0 | 0 | 1 (11.1) |
| Hemorrhoids | 0 | 0 | 1 (11.1) |
| Lip blister | 0 | 0 | 1 (11.1) |
| Respiratory, thoracic, and mediastinal disorders | 0 | 0 | 3 (33.3) |
| Cough | 0 | 0 | 1 (11.1) |
| Dry throat | 0 | 0 | 1 (11.1) |
| Nasal congestion | 0 | 0 | 1 (11.1) |
| Throat irritation | 0 | 0 | 1 (11.1) |
| Nervous system disorders | 0 | 1 (33.3) | 1 (11.1) |
| Dizziness | 0 | 0 | 1 (11.1) |
| Headache | 0 | 1 (33.3) | 0 |
| Skin and subcutaneous tissue disorders | 1 (11.1) | 0 | 0 |
| Dermatitis contact | 1 (11.1) | 0 | 0 |
| Pruritus | 1 (11.1) | 0 | 0 |

Abbreviation: AE, adverse event.
Events that emerged during the combination treatment period.

Supplemental Table S3 Study 2: Summary of AEs by system organ class and preferred term

| **System organ class, n (%) preferred term** | **Single-dose assessment** | | | **Multiple-dose assessment** |
| --- | --- | --- | --- | --- |
|  | **4.5 mg verinurad  (n = 16)** | **6 mg verinurad  (n = 16)** | **12 mg verinurad  (n = 16)** | **12 mg verinurad QD (n = 8)** |
| Gastrointestinal disorders | 0 | 1 (6.3%) | 1 (6.3%) | 1 (12.5%) |
| Diarrhea | 0 | 0 | 1 (6.3%) | 1 (12.5%) |
| Gastrointestinal motility disorder | 0 | 1 (6.3%) | 0 | 0 |
| General disorders and administration site conditions | 0 | 0 | 0 | 1 (12.5%) |
| Feeling cold | 0 | 0 | 0 | 1 (12.5%) |
| Infections and infestations | 0 | 0 | 0 | 1 (12.5%) |
| Rash pustular | 0 | 0 | 0 | 1 (12.5%) |

Abbreviations: AE, adverse event; QD, once daily.

Supplemental Table S4 Study 1: Summary of pharmacokinetic parameters for allopurinol and oxypurinol in healthy Asian and Chinese participants

| **Study day** | **AUC (ng·h/mL)** | **AUC_τ_ (ng·h/mL)** | **CL/F  (L/h)** | **C_max_  (ng/mL)** | **t_max_ (h)** | **t_½_λz  (h)** | **R_ac_ AUC_τ_** | **R_ac_ C_max_** |
| --- | --- | --- | --- | --- | --- | --- | --- | --- |
| Allopurinol: Healthy Asian participants | | | | | | | | |
| Day 1 | 6225 (26.8) | 6226 (26.8) | 49.7 (13.4) | 1773 (35.8) | 2.00 (0.50–3.00) | 1.34 (0.108) | N/A | N/A |
| Day 7 | N/A | 6019 (25.7) | 51.3 (13.7) | 1779 (29.7) | 3.00 (1.00–4.00) | 1.23 (0.0973) | 0.967 (0.791–1.22) | 1.00 (0.578–1.48) |
| Allopurinol: Healthy Chinese participants | | | | | | | | |
| Day 1 | 6206 (25.5) | 6206 (25.5) | 49.7 (12.0) | 1939 (24.7) | 1.52 (0.50–4.00) | 1.43 (0.0853) | N/A | N/A |
| Day 9 | N/A | 5742 (21.0) | 53.3 (11.2) | 1567 (20.1) | 2.55 (1.00–3.10) | 1.40 (0.0891) | 0.932 (0.746–1.17) | 0.787 (0.421–1.14) |
| Oxypurinol: Healthy Asian participants | | | | | | | | |
| Day 1 | N/A | 180700 (18.3) | N/A | 11060 (18.7) | 3.00 (1.50–5.00) | N/A | N/A | N/A |
| Day 7 | N/A | 120200 (18.1) | N/A | 7697 (15.4) | 4.00 (3.00–6.00) | N/A | 0.665 (0.586–0.777) | 0.696 (0.629–0.780) |
| Oxypurinol: Healthy Chinese participants | | | | | | | | |
| Day 1 | 342100 (24.2) | 201400 (18.5) | N/A | 11780 (12.3) | 2.00 (1.50–6.00) | 17.7 (1.98) | N/A | N/A |
| Day 9 | N/A | 139100 (15.7) | N/A | 8520 (10.5) | 3.10 (2.00–6.03) | N/A | 0.687 (0.521–0.824) | 0.720 (0.619–0.805) |

AUC, AUC_τ_, and C_max_, are geometric mean (gCV%); CL/F and t_½_λz are arithmetic mean (SD); t_max_ is median (range); and R_ac_ AUC_τ_ and R_ac_ C_max_ are geometric mean (range).
Abbreviations: AUC, area under plasma concentration-time curve; AUC_τ_, AUC over a dosing interval (24 hours); CL/F, apparent oral clearance; C_max_, maximum observed plasma concentration; gCV%, geometric coefficient of variation; R_ac_, accumulation ratio; SD, standard deviation; t_½_λz, half-life associated with terminal slope of a semi-logarithmic concentration-time curve; t_max_, time to reach C_max_.

Supplemental Table S5 Summary of sUA maximum change from baseline and time of maximum change from baseline in Study 1 and Study 2

|  | **Timepoint** | **Study 1** | | | **Study 2** | | | |
| --- | --- | --- | --- | --- | --- | --- | --- | --- |
|  |  | **Healthy Asian participants** | | **Healthy Chinese participants** | **Single-dose assessment** | | | **Multiple-dose assessment** |
|  |  | **24 mg verinurad + 300 mg allopurinol  (n = 9)** | **Placebo  (n = 3)** | **12 mg verinurad + 300 mg allopurinol (n = 9)** | **4.5 mg verinurad (n = 16)** | **6 mg verinurad  (n = 16)** | **12 mg verinurad (n = 16)** | **12 mg verinurad QD (n = 8)** |
| E_max, CB_, mean (SD), % | Day 1 | –58.3 (8.07) | 4.0 (11.86) | –44.9 (10.81) | –25.8 (6.44) | –29.4 (3.95) | –42.2 (8.11) | –46.5 (7.98) |
|  | Day 7/9 | –73.9 (7.97) | –1.5 (12.34) | –67.2 (7.52) | – | – | – | –70.9 (3.08) |
| t_Emax, CB_, mean (SD), hour | Day 1 | 13.3 (4.00) | 4.0 (3.46) | 11.3 (2.00) | 8.0 (2.1) | 8.0 (2.0) | 9.0 (1.5) | 9.0 (1.0) |
|  | Day 7/9 | 8.7 (6.08) | 2.0 (3.46) | 6.8 (2.12) | – | – | – | 7.0 (3.0) |

Abbreviations: E_max,CB_, maximum observed percentage change from baseline (time-matched, Day –1) in sUA concentrations; QD, once daily; SD, standard deviation, sUA, serum uric acid; t_Emax,CB_, time to E_max,CB_.

Supplemental Figure S1 Study 1: Geometric mean (A) allopurinol and (B) oxypurinol plasma concentration-time profiles in healthy Asian and Chinese participants following single and multiple dosing.

**
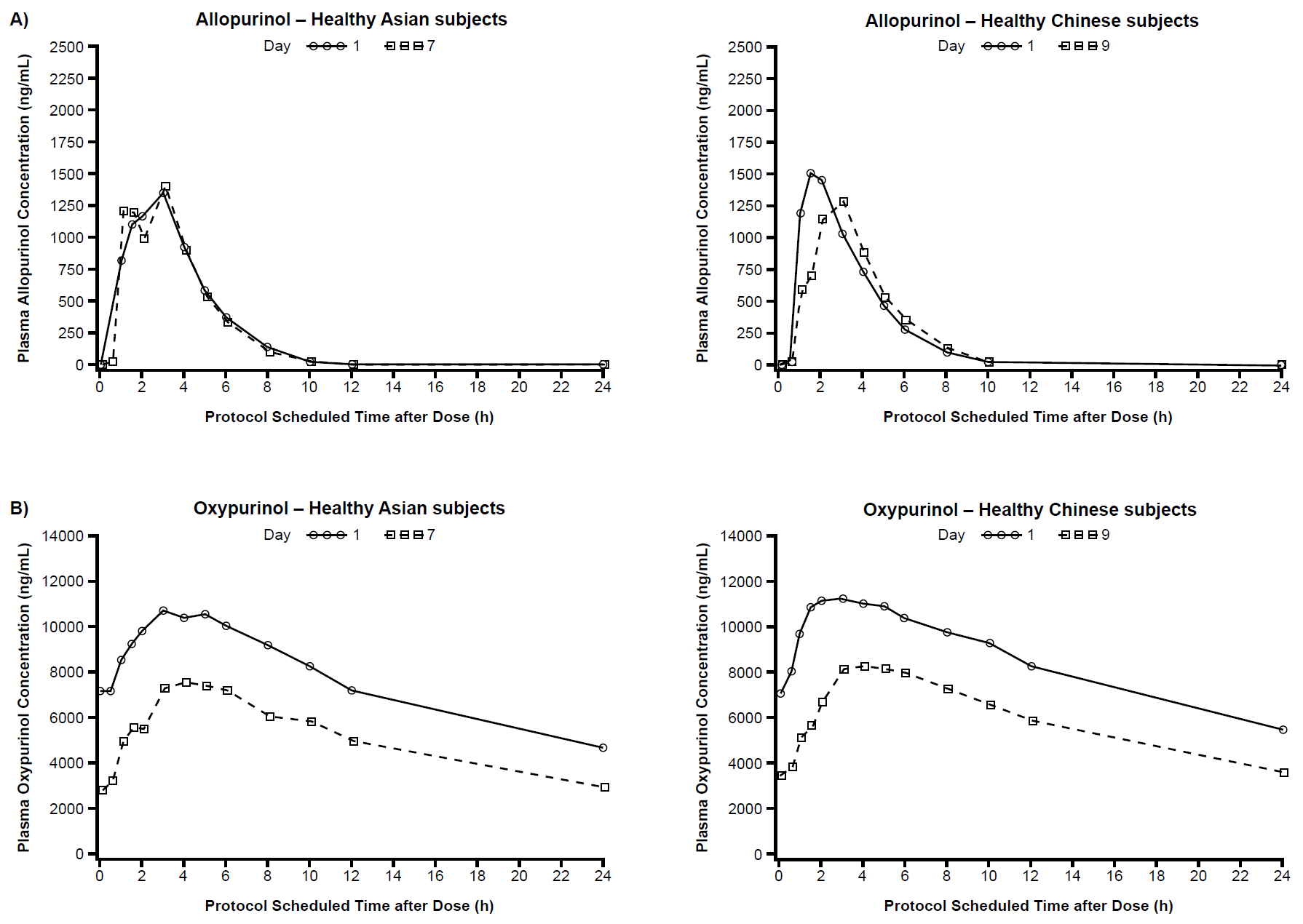
**

Healthy Asian participants: 24 mg verinurad + 300 mg allopurinol once daily for 7 days. Healthy Chinese participants: 12 mg verinurad + 300 mg allopurinol on day 1 and then once daily on days 3–9.

Supplemental Figure S2 Study 1: Mean (SD) time-matched percentage change from baseline (day –1, after run-in period) in Ae_ur_ in healthy Asian and Chinese participants.

Baseline (Day –1) represents Ae_ur_ following 7 days of 300 mg allopurinol during the run-in period.
Abbreviations: Ae_ur_, amount of uric acid recovered in urine; SD, standard deviation

Supplemental Figure S3 Study 2: Mean (SD) time-matched percentage change from baseline (day –1 pre-dose) in Ae_ur_ in healthy non-Asian participants following (A) single and (B) multiple doses of verinurad.

**(A)**

**(B)**

Abbreviations: Ae_ur_, amount of uric acid recovered in urine; QD, once daily; SD, standard deviation.
